# Integrated Quality of Service for Offline and Online Services in Edge Networks via Task Offloading and Service Caching

**DOI:** 10.3390/s24144677

**Published:** 2024-07-18

**Authors:** Chuangqiang Zhan, Shaojie Zheng, Jingyu Chen, Jiachao Liang, Xiaojie Zhou

**Affiliations:** School of Information Engineering, Guangdong University of Technology, Guangzhou 510006, China; 2112103012@mail2.gdut.edu.cn (C.Z.); 2112105186@mail2.gdut.edu.cn (S.Z.); 2112103066@mail2.gdut.edu.cn (J.L.); 2112105143@mail2.gdut.edu.cn (X.Z.)

**Keywords:** online, offline, service caching, inference, mobile edge computing, deep reinforcement learning

## Abstract

Edge servers frequently manage their own offline digital twin (DT) services, in addition to caching online digital twin services. However, current research often overlooks the impact of offline caching services on memory and computation resources, which can hinder the efficiency of online service task processing on edge servers. In this study, we concentrated on service caching and task offloading within a collaborative edge computing system by emphasizing the integrated quality of service (QoS) for both online and offline edge services. We considered the resource usage of both online and offline services, along with incoming online requests. To maximize the overall QoS utility, we established an optimization objective that rewards the throughput of online services while penalizing offline services that miss their soft deadlines. We formulated this as a utility maximization problem, which was proven to be NP-hard. To tackle this complexity, we reframed the optimization problem as a Markov decision process (MDP) and introduced a joint optimization algorithm for service caching and task offloading by leveraging the deep Q-network (DQN). Comprehensive experiments revealed that our algorithm enhanced the utility by at least 14.01% compared with the baseline algorithms.

## 1. Introduction

A digital twin (DT) [1] is a highly promising technology of the modern era that provides a virtual representation of a physical object or system for simulation, analysis, and optimization. In DT deployment, edge servers play a crucial role by enabling the seamless mapping of real-world scenarios through inference and enhancing the intelligence of DT-based services. As DTs become more complex and data intensive, processing large-scale real-time data has become a significant challenge. To address this, artificial intelligence (AI) algorithms applied to DTs [2] have emerged as an effective solution that enable efficient processing of large-scale data and facilitate complex pattern recognition and predictive analysis. Among these AI algorithms, deep reinforcement learning (DRL) algorithms [3] are particularly promising since they continuously enhance the model processing power through automated training and updates. Particularly, a deep Q-network (DQN) is a powerful DRL technique that combines Q-learning with deep neural networks to efficiently handle high-dimensional state spaces. This is crucial for DT services, which often require both online services for real-time applications and offline services for maintaining accuracy in different applications. However, processing data for these diverse services in large-scale networks demands high performance and reliability, posing challenges for centralized DT systems.

To address this issue, multiple-access edge computing (MEC) was proposed as a solution [4]. MEC offers a way to bring cloud computing capabilities and storage to the edge of the network and closer to users, thus reducing the latency and improving the efficiency of processing online service requests. This architecture is particularly beneficial for delay-sensitive and computationally intensive tasks, which makes it a promising approach to handle the challenges posed by the strong coupling between service-caching and task-offloading strategies. It not only facilitates the deployment of computing and resource storage at the network edge but also enables the implementation of diverse algorithms while ensuring the desired levels of the quality of service (QoS). To improve the MEC performance, researchers have developed various algorithms, such as service placement via the randomized rounding algorithm (SPR) [5], which addresses resource constraints and enhances the service deployment efficiency by utilizing randomized rounding techniques to optimize the service placement and evaluate multiple deployment scenarios.

DT and MEC together provide strong support for the digital transformation of the physical world. Previous research made significant strides in this area. Zhou et al. [6] proposed a DT-based resource-scheduling framework to ensure data security in power grid resource scheduling by utilizing Lyapunov optimization for real-time performance. Guo et al. [7] combined deep reinforcement learning with federated learning for allocating edge network resources, thereby enhancing the overall transmission rates. In [8], the authors addressed multi-task interference and reduced the computation delay through model caching and request routing. However, previous research mainly focused on the QoS guarantee for online services [6,7,8]. Ref. [9] introduces a hybrid optimization framework that combines the worst wireless devices adjusting algorithms for wireless power transfer and a DRL model for offloading decisions under time-varying channel conditions and achieved near-optimal total computation delay with reduced computational complexity, which is particularly suited for fast-fading environments. Ref. [10] designed a DRL algorithm to solve the bidirectional preference perception ridesharing problem by maximizing the total utility of passengers while satisfying the constraints of the driver’s buyout asking price, crossing radius, passenger waiting time, budget, and both sides’ detour length, and thus, guaranteeing economic property by incentivizing both passengers and drivers. A critical gap remains: edge servers often handle offline services while processing online services. Online services consume significant computation and memory resources, which can detract from the efficiency of processing offline tasks, thereby impacting the overall QoS performance.

The integration of offline and online services in collaborative edge computing networks for DT applications presents a complex optimization problem. While edge computing offers promising solutions for processing both types of services, achieving an optimal QoS remains challenging due to the diverse nature of these services and the limitations of edge resources. The effective utilization of service caching and task offloading strategies is needed. However, this integration faces two significant challenges:

- **Strong coupling between service-caching and task-offloading strategies:** The interdependence between service-caching and task-offloading strategies makes the problem difficult to solve. For online service requests, computation can only be provided if the corresponding service is cached. Any deviation in the service-caching strategy will trigger changes in the task-offloading strategy to meet the service demands. On the one hand, the service-caching strategy must be precisely configured to fully utilize the server computational power by considering the limited computational capacity of edge servers and the deadline requirements of online tasks. Therefore, service-caching and offloading strategies should be jointly optimized.

- **The conflict between offline and online services’ computational resources:** Offline services strive to utilize as much computational power as is feasible to minimize the latency and boost the response speed, with the aim to provide the highest QoS possible. Conversely, online services require a specific amount of computational resources to meet their strict deadline requirements, thus ensuring timely and reliable service delivery. The disparate QoS performance needs of these two types of services further complicate the process of balancing and optimizing resource allocation, which creates a challenging optimization problem that requires careful consideration and innovative solutions.

To effectively overcome these challenges and address the integrated QoS for the offline and online services problem, our key contributions are as follows:

- **Novel edge service model:** To the best of our knowledge, we were the first to consider the integration requirements of online and offline services for QoS in edge networks for digital twin applications. We propose an innovative edge service model tailored for heterogeneous digital twin services to address this previously overlooked integration. Our model accounts for the diversity of service demands (soft deadlines for offline services and hard deadlines for online services) and the variety of services provided by edge AI providers (such as heterogeneous edge inference devices). We defined a unique optimization objective that balances the reward for online service throughput against penalties for offline services that exceed soft deadlines to effectively reflect the diversity of service demands.

- **Advanced DRL approach:** We introduce a DRL approach that incorporates a DQN to tackle the identified challenges. Our method defines states, actions, and rewards for service-caching and request-routing strategies within a DRL-based service brokerage system. The DQN framework optimizes our objective function while considering key constraints, such as memory and computing resource limitations and hard deadlines. In the search for the optimal action space, we identified and excluded scenarios where services were cached to a server without a corresponding task routing, which prevents the wastage of memory resources.

- **Experimental validation:** Extensive experiments demonstrated the superior performance of our DRL approach, which was characterized by rapid convergence. Compared with traditional baseline algorithms, our method achieved substantial improvements in demand diversity metrics, with average gains of 14.01% and 24.55% in key performance indicators. These results highlight the effectiveness of our DRL-based approach in handling diverse demands and optimizing the system performance.

The remainder of this paper is organized as follows: Section 2 reviews relevant research on QoS for both offline and online services. Section 3 presents an overview of the system model and problem formulation. The proposed DRL algorithm is elaborated in Section 4, while Section 5 delves into the analysis of the experimental results. The paper concludes in Section 6.

## 2. Related Work

### 2.1. QoS of Offline Service

Previous edge-task-offloading research primarily focused on ensuring the QoS for offline services, as shown in Table 1. Ref. [11] considered the hard and soft deadline requirements of tasks in edge networks and designed an offloading strategy to maximize the number of tasks that meet their deadlines. Ref. [12] proposes an approximate algorithm to accommodate the offloading of tasks with mixed deadlines. Ref. [13] introduces a DRL-based task-offloading method to handle real-time task offloading with soft deadlines, with the aim to maximize the real-time task completion ratio. While achieving a high QoS for offline services is crucial, considering online service caching is equally essential to fully utilize the computation resources on edge servers. Ref. [14] focuses on optimizing task offloading in Internet of things (IoT) environments using deep reinforcement learning algorithms employed to maximize resource utilization and meet diverse QoS requirements. Ref. [15] addresses task class partitioning in mobile computation offloading to minimize mobile device power consumption. It employs heuristic class ordering methods based on task latency and mean power consumption criteria, with the aim to meet soft and hard task completion deadlines for offline tasks within specified network resource constraints. Thus, ensuring the QoS for online services must also be well-maintained, especially when both offline and online services coexist on edge servers. For example, solely focusing on ensuring QoS for offline services might result in the remaining server computational resources being inadequate to meet the demands of online services.

### 2.2. QoS of Online Service

Some research on edge task offloading took into account online service caching, but they predominantly concentrated on ensuring QoS for online services, overlooking the QoS demands of offline services. For instance, the study in [16] concurrently planned service-caching and computation-offloading strategies to minimize user network costs within latency limits. In [17], the authors examined service caching and computation resource allocation in edge computing networks and proposed a stochastic service-caching approach to optimize the likelihood of service success. Ref. [18] explores the combined challenge of service caching, computation offloading, and computation resource allocation in multi-user, multi-task environments, with the aim to minimize the overall computation and delay costs for all users. Ref. [19] investigates the challenges of multi-server resource competition and service-caching strategy in a three-tier MEC system. Ref. [20] investigates hierarchical joint caching and resource allocation in cooperative MEC systems using DQN algorithms, with the aim to optimize resource utilization and load balancing between MEC servers. They integrate service caching, resource allocation, and computation offloading using the double-DQN algorithm to optimize latency and energy consumption, with the aim to minimize the overall system cost. Nevertheless, these investigations failed to tackle QoS assurance for offline services.

## 3. System Model

**Figure 1:**
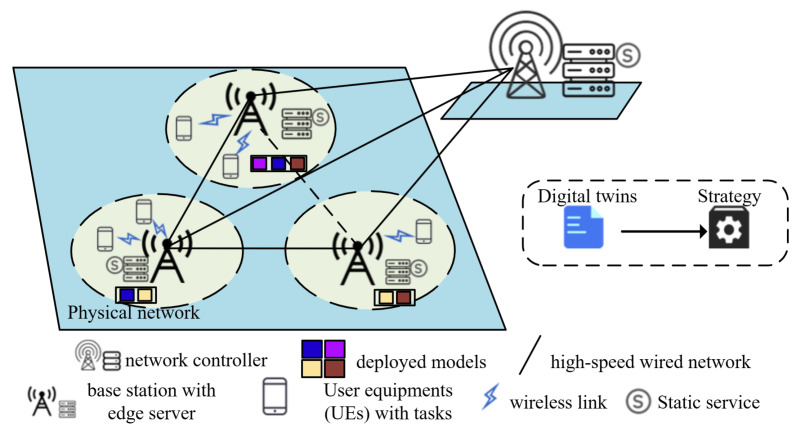
System model of network.

In Figure 1, a set of edge servers is positioned at base stations (BSs) that are specifically denoted as $\Omega =\left\{1,2,3,\cdots \;,S\right\}$. Additionally, there exists a set of user equipment (UE), which is represented as $M=\left\{1,2,3,\cdots \;,M\right\}$. In real-world applications, each piece of user equipment establishes a secure and reliable communication link with a base station via orthogonal frequency division multiple access (OFDMA) technology, as referenced in [8].

The MEC server provides caching and computing services, with the aim to minimize latency. To achieve this, it keeps frequently accessed data close to users by caching virtual digital twins of the UE, BSs, and AI service sub-models. Furthermore, the edge server offers powerful computing capabilities to handle demanding tasks requested by the UE, which significantly reduces the end-to-end latency. The BSs are interconnected by high-speed wired connections, which ensures efficient communication. Within this network architecture, the role of the metaverse service provider is played by either the edge server itself or a centralized controller. This controller oversees the entire system by efficiently allocating processing and storage resources to edge servers and making critical real-time decisions. Additionally, there are *N* AI service sub-models available, which are denoted as $N=\left\{1,2,3,\cdots \;,N\right\}$. Each piece of UE is capable of transmitting crucial data, such as the location, communication details, computational requirements, and service requests, to its designated data terminal. This, in turn, ensures that the global network information within the controlling BS remains up to date. To improve the efficiency, numerous sub-models for AI services can be effortlessly integrated onto the edge server through the utilization of Docker technology. Let the binary variable $z_{m,n}$ represent whether the piece of UE *m* requests model *n* (1) or not (0), as follows:

(1) $$\sum_{n\in N}z_{m,n}=1$$

The linked edge server receives a request from each piece of user equipment for a distinct AI service model, along with the enrollment of its data terminal. Each natural number n∈N corresponds to a quintuple $\left\{c_{n},w_{n},l_{n},o_{n},l_{n}^{soft}\right\}$, where $c_{n}$ denotes the data size, $w_{n}$ denotes the computational resource requirements (GFLOPs) for inference tasks, $l_{n}$ represents the strict delay requirement for the online model, $o_{n}$ denotes whether the model is an offline model (1) or not (0), and $l_{n}^{soft}$ is the soft delay requirement for the offline model.

Let T denote the sequence of time intervals, and let Δt denote the width of each time slot. At the onset of every time slot, a single piece of UE initiates one request. To guarantee the seamless and uninterrupted provision of offline services, we incorporate Δt as a strict delay requirement for offline services. We introduce the binary variable $x_{s,n}\left(t\right)$ to represent whether model *n* is stored in the cache of edge server *s* (1) or not (0). Additionally, we define the binary variable $x_{s,n}^{pre}\left(t\right)$ to signify whether model *n* is an offline service and is pre-cached at edge server *s* (1) or not (0). Considering the restricted primary memory capacity of MEC servers, we obtain the following:

(2) $$\sum_{n\in N}x_{s,n}\left(t\right)\cdot c_{n}\leq R_{s},\forall s\in \Omega$$

where $R_{s}$ denotes the edge server *s*’s available main memory. The binary variable $y_{s,m}\left(t\right)$ indicates whether a request from UE *m* can be offloaded to edge server *s* at time *t*. If the edge server *s* successfully receives and processes the request from UE *m*, it can be formalized as

(3) $$\sum_{s\in \Omega }y_{s,m}\left(t\right)=1,\forall m\in M$$

Tasks can only be offloaded to an MEC server that already possesses the relevant service model. Based on this premise, we draw the following conclusion:

(4) $$y_{s,m}\left(t\right)\cdot z_{m,n}\leq x_{s,n}\left(t\right),\forall s\in \Omega ,m\in M,n\in N$$

Due to the fact that the offline model is pre-cached, we obtain

(5) $$x_{s,n}^{pre}\leq x_{s,n}\left(t\right),\forall s\in \Omega ,n\in N$$

### 3.1. Communication Model

Denote the set of UE connected to BS *s* as $M_{s}$. The rate of data transmission from UE *m* to *s* is represented as

(6) $$r_{s,m}\left(t\right)=W\log_{2}\left(1+\frac{p_{m}\cdot H_{s,m}\left(t\right)}{WN_{0}}\right),\forall s\in \Omega ,m\in M$$

where *W* stands for the bandwidth of the communication channel, while $H_{s,m}\left(t\right)$ signifies the channel gain achieved over the wireless link between UE *m* and BS *s*, and $N_{0}$ denotes the power of the Gaussian noise. The channel gain is provided as

(7) $$H_{s,m}\left(t\right)=D_{s,m}{\left(t\right)}^{-\eta }{\left|h_{s,m}\right|}^{2}$$

where $D_{s,m}$ represents the distance between UE *m* and server *s* in Euclidean space, η was set to 4, and $h_{s,m}$ denotes the coefficient representing signal fading due to Rayleigh scattering in the wireless channel. Particularly, if $m\notin M_{s}$, then $r_{s,m}\left(t\right)=0$. To calculate the delay incurred when offloading UE *m*’s request to server s, the following formula is used:

(8) $$t_{s,m}^{tr}\left(t\right)=\frac{\sum_{n\in N}z_{m,n}d_{n}}{r_{s_{m},m}\left(t\right)}+\sum_{e_{i,j}\in E\left(s_{m},s\right)}\left(\frac{\sum_{n\in N}z_{m,n}d_{n}}{v_{i,j}}+\rho_{i,j}\right)$$

where $d_{n}$ is the normalized input data size for the request of model *n* that originates from UE *m*, $s_{m}$ identifies the BS or server associated with UE *m*, and $v_{i,j}$ denotes the wired data transfer rate between MEC server *i* and server *j*. Additionally, E(i,j) comprises the set of connections that form the most direct route from edge server *i* to server *j*, potentially involving multiple relays, with the condition that *i* and *j* are not equal to $s_{m}$. If the computational task of UE *m* is delegated to $s_{m}$, then $E(s_{m},s)$ is considered an empty set. The propagation delay [6] is $\rho_{i,j}$, i,j∈Ω, and was set as 0.5 ms.

### 3.2. Computational Framework

The computation delay caused by a request from UE *m* decreases as the assigned computing resources, denoted as $f_{s,m}\left(t\right)$ and measured in GFLOPs, increases. This relationship is formally defined as follows:

(9) $$t_{s,m}^{com}\left(t\right)=\frac{\sum_{n\in N}\cdot z_{m,n}\cdot w_{n}}{f_{s,m}\left(t\right)},\forall s\in \Omega ,m\in M$$

Incorporating the computing resource allotment $C_{s}$ of the MEC servers, we formulate the subsequent limitation:

(10) $$\sum_{m\in M}y_{s,m}\left(t\right)\cdot f_{s,m}\left(t\right)\leq C_{s},\forall s\in \Omega$$

Hence, the overall time required to complete a request can be expressed as the summation of both the transmission and computation delays:

(11) $$t_{s,m}\left(t\right)=t_{s,m}^{tr}\left(t\right)+t_{s,m}^{com}\left(t\right),\forall s\in \Omega ,m\in M$$

### 3.3. Problem Definition

Our primary goal was to ensure high-quality service for both online and offline tasks. To achieve this, we defined utility functions as the total throughput minus the sum of the penalties incurred by offline tasks due to timing out while considering diverse limitations, including the delay, computation, and storage constraints. Define the binary variable $\tau_{m}\left(t\right)$ as an indicator of whether the request from UE *m* is completed before its hard delay deadline (1) or not (0). Hence, we obtain

(12) $$\tau_{m}\left(t\right)=\left\{\begin{array}{cc} 1 & \mathrm{if}\sum_{s\in \Omega }y_{s,m}\left(t\right)\cdot t_{s,m}\left(t\right)\leq \sum_{n\in N}z_{m,n}\cdot l_{n}.\\ 0 & \mathrm{otherwise}. \end{array}\right.$$

Let $\varphi_{m}\left(t\right)$ represent the offline task timeout penalty for exceeding the soft deadline; hence, we obtain

(13) $$\varphi_{m}\left(t\right)=\max\left\{\sum_{n\in N}\sum_{s\in \Omega }z_{m,n}o_{n}\cdot \frac{t_{s,m}\left(t\right)-l_{n}^{soft}}{l_{n}^{soft}},0\right\}$$

Tasks that are rejected can be rerouted to a distant or cloud-based server, potentially resulting in greater financial expenses, but providing the advantage of decreased latency expenses thanks to a superior bandwidth. This aspect will be further explored in our future investigations. To ensure the QoS for both online and offline tasks, problem P is expressed as

(14) $$\begin{array}{c} obj:\left(P\right)\max\sum_{t\in T}\sum_{m\in M}\left(\tau_{m}\left(t\right)-\varphi_{m}\left(t\right)\right)\\ s.t.(2)∼(10)\\ x_{s,n}\left(t\right),y_{s,m}\left(t\right)\in \left\{0,1\right\},\forall s\in \Omega ,m\in M,n\in N \end{array}$$

where (2) and (10) denote the primary memory and computing resource limits of the edge servers, respectively. Equation (3) ensures that each piece of UE is served exclusively by one edge server, while (4) verifies that a UE request can be solely delegated to an edge server equipped with the corresponding model. Ultimately, (5) confirms the pre-caching of offline models.

**Theorem** **1.** *Problem P is NP-hard.*

**Proof** **of** **Theorem** **1.** We establish the NP-hardness of the proposed problem by demonstrating a reduction from the renowned NP-hard multi-knapsack problem (MKP) to a specialized case of our own problem, thereby proving its computational complexity. Consider a distinct scenario where there exists only a single edge server, and no offline models are pre-cached. Instead, all models are cached for online services. In this context, we can draw parallels between the computing resource of the edge server and the capacity of a knapsack. In this scenario, each type of request can be treated as an item in the set I, with its value representing the number of requests of that type. The corresponding computing resource required for processing each request can be seen as the fractional size of the knapsack. By establishing this mapping, we relate the original problem to this special case. As the formulated problem represents a specialized instance with specific constraints, it can be inferred that solving the original problem P is even more challenging. Given that the MKP is known to be NP-hard and can be reduced to our specific case, it follows that our proposed problem is also NP-hard.   □

### 3.4. Problem Reformulation

Next, we incorporate an additional variable $f_{s,m}^{on}\left(t\right)$ to represent the minimal computational resource required for servicing an online task of type *n* to fulfill the delay constraint of UE *m*. This variable is defined as follows:

(15) $$f_{s,m}^{on}\left(t\right)=\frac{\sum_{n\in N}z_{m,n}\cdot w_{n}}{\sum_{n\in N}z_{m,n}\cdot l_{n}-t_{s_{m},s}^{tr}\left(t\right)},\forall s\in \Omega ,m\in M$$

To accommodate the maximum number of online pieces of UE, the computing resource allocated to each piece of UE with online tasks by the corresponding edge server is set to $f_{s,m}^{on}\left(t\right)$. The computing resource each piece of UE with offline tasks has is set to $f_{s,m}^{off}\left(t\right)$; thus,

(16) $$\sum_{s\in \Omega }y_{s,m}\left(t\right)\leq 1,\forall m\in M$$

(17) $$\sum_{m\in M}y_{s,m}\left(t\right)\cdot \left(\left(1-o_{n}\right)\cdot f_{s,m}^{on}\left(t\right)+o_{n}\cdot f_{s,m}^{off}\left(t\right)\right)\leq C_{s},\forall s\in \Omega$$

Simultaneously, $\tau_{m}\left(t\right)$ can be alternatively expressed as

(18) $$\tau_{m}\left(t\right)=\sum_{s\in \Omega }y_{s,m}\left(t\right)$$

Thus, we obtain (P1):

(19) $$\begin{array}{c} obj:\left(P1\right)\max\sum_{t\in T}\sum_{m\in M}\left(\tau_{m}\left(t\right)-\varphi_{m}\left(t\right)\right)\\ s.t.(2)∼(5),(16),(17)\\ x_{s,n}\left(t\right),y_{s,m}\left(t\right)\in \left\{0,1\right\},\forall s\in \Omega ,m\in M,n\in N \end{array}$$

Problem (P1) is defined by the objective function that maximizes the throughput of online services while considering the penalty for offline services that exceed their soft deadline, which aligns with the integrated QoS utility metric we established. Given the complexity of this problem, it necessitates the development of a new algorithm for its resolution.

## 4. Algorithm and Analysis

### 4.1. Deep Reinforcement Learning Algorithm

**Figure 2:**
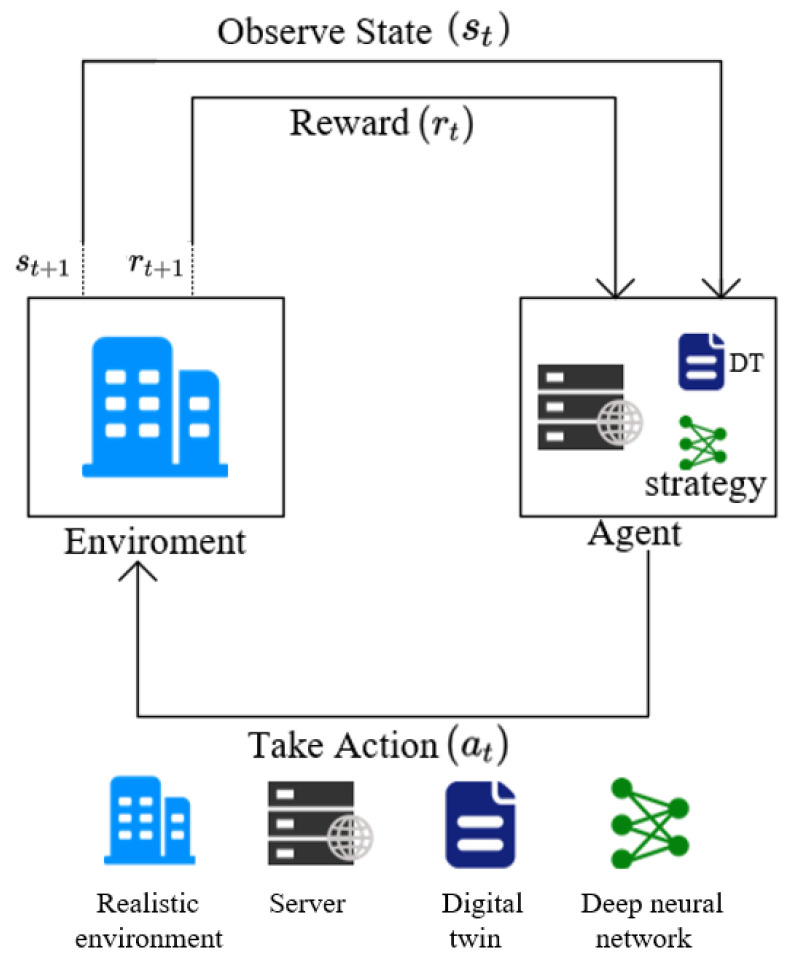
Perception–action–learning framework.

When addressing issues related to model caching and task offloading, they can be naturally formulated as serialized Markov decision processes (MDPs) due to the fact that the next state is determined solely by the current state and the action taken. Within this part of our discussion, we showcase a DRL algorithm that incorporates the DQN approach. Initially, we define the states, actions, and rewards associated with the DRL-based service caching and request routing matching policy. States typically represent the current system configuration and resource utilization; actions refer to various strategies that can be adopted by the system, such as caching a model or offloading a task; while rewards are defined based on the objectives of the system, such as the response time and resource utilization. Subsequently, we leverage the DQN framework to optimize our objective function while taking into account crucial constraints, like memory resource constraints, computing resource constraints, and hard deadlines. DQN approximates the Q-value function using a neural network, which enables it to handle large state spaces and complex decision-making processes. By incorporating these constraints into our optimization process, we aimed to achieve a more intelligent and efficient service caching and request routing matching policy that maximizes the system performance while satisfying critical resource and timing requirements. The operational framework of the algorithm is shown in Figure 2. The agent observes the current state $s_{t}$ and receives a reward $r_{t}$ from the environment, and then takes an action $a_{t}$ based on its strategy. This action affects the environment, leading to a new state $s_{t+1}$ and a new reward $r_{t+1}$. This process continues iteratively, which allows the agent to learn and optimize its decision making over time.

- State: The state of the environment is represented by a state variable in the framework, and this state has a direct influence on the reward feedback that is associated with the agent’s actions within the system. Define *K* as the overall count of the time steps, with *k* indicating the specific *k*-th step within this sequence, where 1⩽k⩽K. $s_{k}=\left\{{\hat{y}}_{s,m}\left(k\right),{\hat{x}}_{s,n}\left(k\right),s_{m}\left(k\right),p_{s,n}\left(k\right),x_{s,n}^{pre}\left(k\right),z_{m,n}\left(k\right),{\hat{C}}_{s}\left(k\right),{\hat{R}}_{s}\left(k\right)\right\}$ is the state vector of the system at the *k*-th time step. ${\hat{y}}_{s,m}\left(k\right)$ and ${\hat{x}}_{s,n}\left(k\right)$ represent the task-offloading strategy and the model-caching strategy at the *k*-th time step, respectively. The variables $s_{m}\left(k\right)$ and $p_{s,n}\left(k\right)$ represent the connection status between the piece of UE and the base station, as well as the distance between the piece of UE and the connected base station. On the other hand, $x_{s,n}^{pre}\left(k\right)$ and $z_{m,n}\left(k\right)$ indicate whether the static model *n* is cached on server *s* or not, and whether the dynamic and static task from user device *m* requests model *n* or not, respectively. The remaining storage capacity and computing resources at time step *k* are represented as ${\hat{C}}_{s}\left(k\right)$ and ${\hat{R}}_{s}\left(k\right)$. For every event, the state vector is set to the zero vector as its initial state.

- Action: The action vector for the *k*-th time step can be expressed as $\left\{{\tilde{y}}_{s,m}\left(k\right),{\tilde{x}}_{s,n}\left(k\right)\right\}$, where ${\tilde{y}}_{s,m}\left(k\right)$ indicates that the task of the user device *m* is offloaded to the server *s* and ${\tilde{x}}_{s,n}\left(k\right)$ indicates that the model *n* is cached on the server *s*.

- Reward: In DRL, the network controller acquires knowledge through rewards. The objective of the network controller is to maximize the total utility provided by both dynamic and static services. To guide the network controller toward the optimal solution for the target task, the reward $r_{k}$ is formulated as the disparity in the utility of the user device following the execution of the action $a_{k}$ compared with before taking action $a_{k}$ and can be represented as

(20) $$r_{k}=U^{\prime }\left(k\right)-U\left(k\right)$$

The utilities $U^{\prime }\left(k\right)$ and U(k) denote the overall utilities of the dynamic and static services after and before executing action $a_{k}$, respectively. $U^{\prime }\left(k\right)$ is computed by applying the current action $a_{k}$ given the state $s_{k}$. It is crucial to mention that if any QoS constraints are violated upon executing action $a_{k}$, the reward is penalized by setting it to -*∞* and the event is immediately concluded.

Management of the large state space of $s_{k}$ using only a predefined Q-table to query and update Q-values for each state–action pair is insufficient. Therefore, DQNs are used to predict Q-values, which effectively addresses this issue.

The Q-function, which is denoted as $Q(s_{k},a_{k})$, represents the expected reward for executing action $a_{k}$ in state $s_{k}$. The DRL algorithm utilizes the temporal difference method to iteratively update the Q-function and adjust its values based on the differences between the predicted and actual rewards, as reflected in the equation below:

(21) $$\begin{array}{c} \begin{array}{cc} Q(s_{k},a_{k}) & =Q\left(s_{k},a_{k}\right)+\gamma \left[r_{k}+\eta \max_{a_{k}\in A}Q\left(s_{k+1},a_{k+1}\right)-Q\left(s_{k},a_{k}\right)\right] \end{array} \end{array}$$

The learning rate γ and discount factor η fall within the ranges 0 < γ < 1 and 0 < η < 1. In DRL, the ϵ-greedy strategy [21] is employed. Due to the large state space in our problem, using only a predefined Q-table to query and update the Q-values is inadequate. The DQN resolves this issue by utilizing a neural network to approximate the Q-value for a given state–action pair (*s*, *a*), thereby superseding the traditional Q-table approach employed in Q-learning. During the training process, this neural network undergoes iterative updates. The DQN adjusts the neural network’s weight parameters at every iteration *i*, with the aim to minimize the loss function that follows:

(22) $$\begin{array}{c} L_{i}\left(\theta_{i}\right)=E\left[{\left(y_{i}-Q\left(s,a\;;\theta_{i}\right)\right)}^{2}\right] \end{array}$$

In this context, $y_{i}$ represents the expected return, which is calculated using the formula $y_{i}=E\left[r+\eta \max_{a^{\prime }}Q\left(s^{\prime },a^{\prime };\theta_{i-1}\right)\right]$. This expectation is derived from the target Q-value estimator $\hat{Q}$, which employs the same neural network structure as the Q-network. The action $a^{\prime }$ considered here is taken from the state s based on the previous set of weights $\theta_{i-1}$ and leads to the new state $s^{\prime }$. η = 0.9 was the discount factor that was selected. Based on (22), and using the gradient descent method, the gradients of the weights for the Q-network can be updated according to the following formula:

(23) $$\begin{array}{cc} \; & \nabla_{\theta_{i}}L_{i}\left(\theta_{i}\right)=E\left[{\left(r+\eta \max_{a^{\prime }}Q\left(s^{\prime },a^{\prime };\theta_{i-1}\right)-Q\left(s,a\;;\theta_{i}\right)\right)}^{2}\right]\cdot \nabla_{\theta_{i}}Q\left(s,a\;;\theta_{i}\right) \end{array}$$

Algorithm 1 provides a detailed description of the DRL process. First, the evaluation network’s and target network’s settings are set up and the replay memory’s capacity is set. The initial state *s* is set at the start of every episode. Next, the agent uses the ϵ-greedy strategy to choose an action $a_{k}$ at each time step *t*. Following the execution of the selected action $a_{k}$, the DRL receives the associated reward $r_{k}$ and the ensuing state $s_{k+1}$. In the replay memory designated as *D*, these data are kept as a transition ($s_{k}$, $a_{k}$, $r_{k}$, $s_{k+1}$). Later, the evaluation network’s parameters are modified by randomly selecting a mini-batch of transitions from *D*. Additionally, the parameters of the target network are modified to match those of the evaluation network every *C* steps (*C* = 200). After the DRL algorithm is executed, any tasks that exceed their hard deadline are offloaded to the cloud for processing. This ensures that tasks that cannot be completed within the specified time constraints on the local system are handled by the cloud to leverage its greater computational resources to meet the necessary deadlines.
**Algorithm 1** DRL: Deep Reinforcement Learning  1:Initialize replay memory *D* with a certain capacity of $10^{4}$. Initialize the *Q* function with random weights θ. ReLU is the activation function.  2:**for** per episode **do**  3:   Initialize the state sequence $s_{1}$.  4:   **for** each time step *k*← 1 to *K* **do**  5:     Examine the present network state $s_{k}$.  6:     Choose a random action $a_{k}$ with ϵ-greedy strategy.  7:     Run the emulator and execute action $a_{k}$, then notice reward $r_{k}$ and the new network state $s_{k+1}$.  8:     **if** $r_{k}\left(s_{k},a_{k}\right)$ = -*∞* **then**  9:        Break.10:     **end if**11:     Store transition ($s_{k}$, $a_{k}$, $r_{k}\left(s_{k},a_{k}\right)$, $s_{k+1}$) into memory *D*.12:     Sample a random mini-batch of transitions *D*’ from *D*.13:     $y_{k}\leftarrow r_{k}\left(s_{k},a_{k}\right)+\eta \max_{a\in A}\hat{Q}\left(s_{k+1},a^{\prime };\theta^{\prime }\right)$, where $\hat{Q}$ is estimated by the target network.14:     Refresh the evaluation network parameters θ by executing the gradient descent using (23).15:     Set $\hat{Q}$ = *Q* every *C* steps (*C* was set as 200 in our experiment).16:   **end if**17:**end for**

### 4.2. Complexity Analysis

In this section, we delve deeper into the intricacies of the DQN-based algorithmic framework, particularly emphasizing its complexity analysis. At the heart of this approach lies the utilization of a DNN to approximate the Q-value function within the Q-learning paradigm. The primary driver of temporal complexity in this setup is rooted in the DNN training phase, which spans across *S* episodes, with each consisting of *T* time steps, which contributes to a foundational complexity of O(ST). Within each time step, the intricacy associated with DNN training can be meticulously gauged by aggregating the multiplicative interactions between consecutive layer dimensions. This is formally expressed as $O(L_{0}\cdot L_{1}+L_{1}\cdot L_{2}+\cdot \cdot \cdot +L_{n}-1\cdot L_{n})$, where $L_{i}$ denotes the input dimension of layer *i*. Notably, the dimension of the initial layer $L_{0}$ is determined by the interplay of the batch size *B* and the state space dimensionality *R*, which renders $L_{0}=BR$. Conversely, the output layer’s dimension is contingent upon the size of the action space A. Therefore, the overall time complexity of the proposed algorithm can be succinctly articulated as $O(ST\times \left(BR\cdot L_{1}+L_{1}\cdot L_{2}+L_{2}\cdot L_{3}+L_{3}\cdot L_{4}+L_{4}\cdot A\right))$, which encapsulates the intricate interplay between the number of episodes and time steps, as well as the intricate computations within the DNN architecture.

## 5. Performance Evaluation

The experiments were conducted on an Intel(R) i9-12900KF workstation to measure the inference time of six AI network models. These models, which were tailored for AI inference services, simulate tasks such as traffic flow prediction and natural disaster risk assessment within digital twin applications. The size of the input for these models remained constant at 224 px × 224 px × 3. The pieces of UE were connected to edge servers via small base stations within a range of [25,80] m. The computing capacities of the edge servers were confined to the range of 100 GFLOPs to 130 GFLOPs, while their storage capacities were limited to [800,1000] MB. Each server had a connection probability of 0.6 to other servers via wired high-speed optical fiber. Storage resources for caching services were determined by the model size, while computing resources for one inference were measured using thop [8]. Table 2 provides detailed notation explanations. The reported data in the figure curves represent the averages over 100 repeated runs across various baseline algorithms.

- SPR-Fix (SPRF): We introduce SPR-Fix, which is a modification of the SPR algorithm [5] that jointly optimizes service caching and request routing to maximize the total system throughput. This variant of SPR incorporates fixed model-caching strategies to represent static model deployment constraints, as outlined in (5). To ensure compliance with the edge server’s constraints regarding primary memory capacity and computing resources, SPR-Fix systematically eliminates overloaded services and task requests to maintain optimal system performance. The removal process follows an ascending order determined by the main memory and computing resource requirements until all limitations are satisfied.
- SPR-Fix + Greedy (SPRFG): Expanding upon the SPR-Fix framework, SRPFG integrates a greedy algorithm to enhance the throughput within the collaboration network. This algorithm prioritizes the offloading of unsatisfied tasks that demand minimal computing resources to edge servers in a greedy fashion.

**Figure 3:**
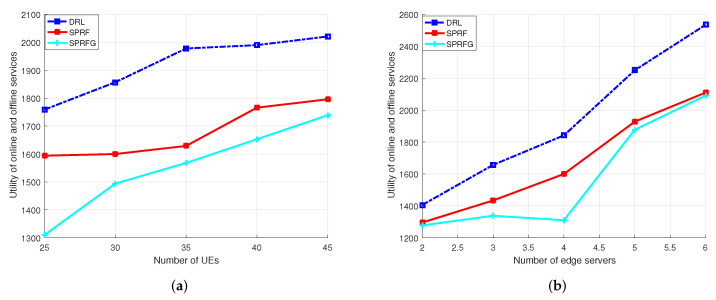
Integrated QoS utility with regard to (**a**) the number of pieces of UE and (**b**) the number of edge servers.

Figure 3a illustrates that with four edge servers, the integrated QoS utility rose alongside the increase in users. With an increasing number of pieces of UE, the reward for the throughput of online tasks increased more than the penalty for offline services that exceeded their soft deadlines, which led to an improvement in the integrated QoS utility. However, the DRL algorithms outperformed the baseline algorithms in terms of performance. On average, DRL demonstrated 14.01% and 24.55% enhancements in utility for online and offline services in heterogeneous-type inference applications compared with SPRF and SPRG, respectively. In Figure 3b, the shifts in integrated QoS utility of the server are depicted with a fixed user count of 35. This trend occurred because a higher number of edge servers provided users with more computing and storage resources. On average, DRL outperformed SPRF by 15.39% and SPRG by 23.13%.

**Figure 4:**
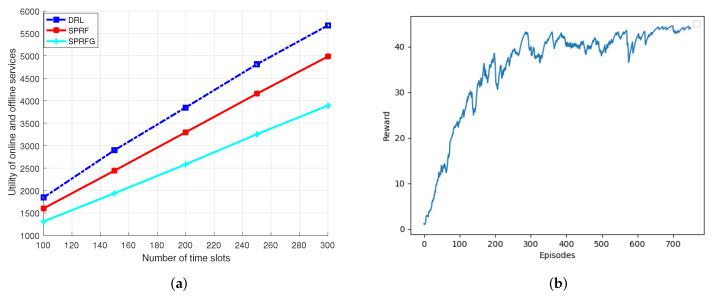
(**a**) Integrated QoS utility with number of time slots and (**b**) training convergence trend.

In Figure 4a, when the number of time slots was set to 100, each with a fixed duration of 0.5s, the integrated QoS utility escalated with an increase in the time slots. To assess the algorithm’s performance, we confirmed its stable operation as the number of time slots increased. Furthermore, under continuous and stable operation, the algorithm also led to a gradual increase in the integrated QoS utility. Notably, as the operation time was extended, the DRL algorithm consistently outperformed the baseline algorithm. DRL exhibited average superiorities of 15.83% over SPRF and 46.51% over SPRG. As depicted in Figure 4b, it is evident that in the initial stages of training for DRL, the integrated QoS utility was below its stable value. This was due to the fact that the DRL agent had not yet learned sufficient information to make optimal decisions regarding the allocation of computational and memory resources. However, with an increased number of episodes, the overall integrated QoS utility progressively settled and approached a steady level.

## 6. Conclusions

In this study, we introduced a novel DT framework that was designed to facilitate heterogeneous interaction in AI applications through collaborative MEC. Our research addressed the critical challenges of service caching and task offloading in collaborative edge networks, with the aim of ensuring integrated QoS for both offline and online services. To tackle these challenges, we developed a DQN-based DRL algorithm. This algorithm was specifically designed to aid in decision making for online service caching and task offloading. We defined an integrated QoS metric as the reward function, which was calculated by subtracting the penalty for offline services that exceed their soft deadlines from the throughput of online services. The experimental evaluations demonstrated that our proposed algorithm significantly outperformed the baseline algorithms in terms of the integrated QoS utility, with average gains of 14.01% and 24.55%. However, we acknowledge the limitations in our current study, which also point to directions for future work. Due to practical constraints, we did not fully explore the impacts of wireless bandwidth allocation on task completion efficiency and overall QoS optimization. Our model also primarily focuses on resource allocation based on average network conditions, without fully addressing dynamic changes in task transmission rates and channel gains. These limitations present opportunities for further research and improvement. To address these challenges and extend the scope of our work, we propose the following directions for future investigation:

- **Enhancing dynamic adaptability:** incorporate real-time bandwidth allocation as a key decision variable to improve the system responsiveness to fluctuating network conditions.
- **Addressing time-varying network conditions:** develop a multi-timescale decision-making process that combines long-term strategies with short-term adjustments for better adaptation to dynamic environments.
- **Exploring advanced DRL algorithms:** integrate and compare more advanced algorithms, such as DDPG and TD3, with our current approach to identify the most effective techniques for complex edge computing scenarios in DT applications.
- **Scaling to larger networks:** evaluate the scalability and robustness of our approach in larger-scale edge computing networks to ensure practical applicability in real-world DT deployments.
- **Optimizing energy efficiency:** incorporate energy consumption considerations into our model to develop a more comprehensive QoS metric that balances performance with sustainability.

By addressing these aspects, we aim to enhance the efficiency, adaptability, and practical applicability of our approach in real-world, time-varying network environments for DT applications. This work provides a foundation for optimizing integrated QoS in collaborative edge networks and opens new avenues for advancing edge computing in DT applications.

## Figures and Tables

**Table 1:** Summary of differences for QoS-oriented works offering offline or online services.

References	Memory Resource Constraint	Computation Resource Constraint	Collaborative MEC Devices	QoS for Offline Service	QoS for Online Service
He et al. [11]	×	✓	×	✓	×
He et al. [12]	×	✓	×	✓	×
Huang et al. [13]	×	✓	×	✓	×
Jain et al. [14]	×	✓	×	✓	×
Chen et al. [15]	×	✓	×	✓	×
Hao et al. [16]	×	✓	✓	×	✓
Kim et al. [17]	✓	✓	×	×	✓
Zhang et al. [18]	✓	✓	×	×	✓
Wang et al. [19]	✓	✓	×	×	✓
Zhang et al. [20]	✓	✓	×	×	✓
Our work	✓	✓	✓	✓	✓

**Table 2:** Default parameters utilized for simulation.

Parameters	Values
Duration of time slot	0.5 s
Number of time slots	100
Number of pieces of UE	35
Number of MEC servers	4
Computing capacity of each edge server	100∼130 GFLOPs/s
Storage capacity per edge server	800∼1000 M
Distance between an MEC server and piece of UE	25∼80 m
Bandwidth of each piece of UE	5 MHz
Transmission power of each piece of UE	25 dBm
Gaussian noise power $N_{0}$	−174 dBm/Hz
Rayleigh fading channel coefficient $h_{s,m}$	1
Wired fiber-optic transmission rate	100 Mbps
Maximum task deadline	400 ms

## Data Availability

Data are contained within the article.
